# Extra Supplement—The Nursing Mirror

**Published:** 1890-11-08

**Authors:** 


### "The Hospital" Nursing Mirror.

Being the Extra Nursing Supplement of "The Hospital" Newspaper.

All Contributions for this Supplement should be addressed to the Editor, The Hospital, 140, Strand, London, W.C., and should have the word "Nursing" plainly written in left-hand top corner of the envelope.

### En Passant.

SICK NURSES.—If any nurse is in need of petting and care for a week to bring her back to health after a long illness, she can secure it (for 10s. 6d.) at a nice home we know kept by two sisters. But it is not a holiday home; it is really for a tired-out nurse. Further particulars can be had from Sister Hilda, Witney, Oxon.

ASYLUM ATTENDANTS.—A controversy has been going on in the *British Medical Journal* on the subject of the status and training of asylum attendants. Dr. Farrar called them "a very low class of girls," but others have come forward to speak their praises, notably the senior assistant medical officer of the Hampshire County Asylum. The fact is that in a few of the asylums, the attendants are *not* properly trained, though in most of them they have lectures and are properly cared for. Each doctor ought not to judge by his own asylum, or by two or three asylums, but try to get a full grasp of the subject. It is all very well to go over a pretty private asylum like one we know, where the nurses receive lectures and excellent salaries, are all well-educated and bright, and attired in the most picturesque and original of uniforms. And some county asylums, where the medical officer is interested in the attendants and has the help of a good matron, give excellent training. But there is also the dark side of the picture which shows asylums where dismissed domestic servants, and raw village girls, untrained even in household work and cleanliness, are accepted. There are no lectures in these asylums; experience is picked up by those who are sharp, but training there is none. And the sick wards of these asylums are under these untrained attendants.

ENGLISH NURSES IN EGYPT.—The following paragraph has been going the round of the daily papers: "In 1889 the Khedive, after much persuasion, consente to the employment of three English certificated nurses on the female side of the Kasr-el-Aini Hospital at Cairo, and the Egyptian Government made a grant for their salaries, maintenance, and passage money. Their work was found so useful in teaching and training the native nurses that the number was increased in 1889, as the Khedive had consented to the English nurses taking charge of the male as well as the female side of the hospital. This was a great triumph over Mussulman prejudices, the value of which in the future it is difficult to estimate. A subscription was opened in London and Cairo to further increase the number of nurses in order that some of them might be always available for nursing private cases of illness. Two have been recently selected from the trained nursing staff of St. Bartholomew's Hospital, London, and will be available in the winter season 1890-91. Up to the present time the nurses have been rather indifferently lodged in apartments within the Kasr-el-Aini Hospital. Owing to the death of Miss Hughes, one of the nurses, from typhoid fever, caught in the performance of her arduous duties, a kind and generous friend, much interested in her and in the work, subscribed a large sum for the erection of a separate building as a home for the nurses, in a more healthy position outside the hospital. The new home is to be called the 'Marian Hughes Nursing Home,' in commemoration of the services of a noble woman who sacrificed her life for the safety of others. The Egyptian Government supplemented the gift, and it is hoped that the building will be ready for occupation early in 1891."

THE LONDON HOSPITAL.—The following resolution, which was unanimously passed at a meeting of the Medical and Surgical Staff of this Hospital, shows the admirable results of the nursing arrangements at the London Hospital: "That the Medical Council having regard to the recent proceedings before the Committee of the House of Lords, desires to convey to the Matron, Miss Lückes, its full sympathy with her on the occasion; its confidence in the management of her department; and its grateful appreciation of her successful efforts to improve the character of the nursing." The Medical and Surgical Staff have done well to thus record the verdict, not only of medical men and laymen throughout the hospital world, but of the general public also, who have come to see the interested motives of the small clique who have so signally failed in their attempt to injure our greatest hospital.

NEWS FROM RUGBY.—An interesting report has been issued by the Rugby District Nursing Association, which specially prides itself on having secured the services of Miss Reid, who trained for twelve years at the Prince Alfred Hospital at Sydney. Australian readers please note. At the annual meeting the Rev. J. Murray proposed that the association be affiliated with the Queen's Institute. The conditions had already been referred to in the report, and he thought, when they bore in mind the noble and munificent gift that had been made by our Queen to the country, and that the women's offering at the jubilee, amounting to £72,538, had been presented by her for the benefit of her suffering countrywomen and the suffering poor of the realm, they would see that it was really a call upon them to co-operate with Her Gracious Majesty in giving effect to her noble generosity. There were benefits arising from it which would result to themselves, and they would have the advantage of co-operating in a national work, and would have the benefit of the experience which was gained in a larger sphere than their own. The resolution was adopted.

SHORT ITEMS.—The *Londonderry Sentinel* for October 28th had a very sensible article on Trained Nurses for Workhouses, showing that some Irish editors appreciate the seriousness of this question.—The East London Nursing Society is issuing a special appeal for help, which concludes by quoting as "a Royal example" the founding of the Queen Victoria Jubilee Institute.—Miss Elizabeth Feachem, late of St. Raphael's and the Levick Institution, is going to start a nursing institute at Worthing. We should like further particulars.—This month's *Girl's Own Paper* contains a portrait and notice of Miss Kate Marsden, and a pretty story of two old ladies by "La Petite."—The Primate's eldest daughter, Miss Helen Benson, age 25, has died from diphtheria contracted while visiting a sick employé of her father's.—*Work and Leisure* for this month contains an article on "Cottage Nurses."—The Marquis of Hertford lately opened the Orthopædic Hospital at Birmingham; the matron is Miss Burgess, who has trained under Miss Gibson at the infirmary. There is a lady dispenser at the Orthopædic.—Can any of our readers tell us if it is true that there is a home at Liverpool where the day nurses are on duty from seven a.m. till nine p.m., and the night nurses from nine p.m. till ten a.m.?—A correspondent suggests that we should start a black list for matrons, but we do not see the necessity. A bad matron cannot keep her nurses, and trouble soon ensues.—The *Illustrated English Magazine* has an article on Maria Josepha, Duchess of Bavaria, who acts as a surgical nurse in her husband's celebrated eye hospitals.

### Lectures on Surgical Ward Work and Nursing.

By Alexander Miles, M.B. (Edin.), C.M., F.R.C.S.E.

### Lecture II.—PREVENTING SEPSIS.

But you may be ready to ask, "Why all this talk about sepsis, germs, antiseptics?" "What harm is there in a wound becoming septic?" It is well that you ask this, and you must not rest contented till you have received a satisfactory answer. The importance of keeping your wounds aseptic cannot be exaggerated. It is the difference between successful and unsuccessful surgery; it may be the difference between the life and death of your patient, and remember that in many cases it lies with you to see that they *are* aseptic.

I cannot here and now go into the pathology of all the conditions which are included under the heading of "septic diseases," but when I mention to you that such conditions as septicæmia, pyæmia, erysipelas, hospital gangrene, malignant pustule, are all directly caused by micro-organisms gaining access to wounds, you will be convinced that in keeping your wounds aseptic you are warding off diseases which your experience teaches are to be dreaded alike by the patient, the surgeon, and the nurse.

As we go on with these lectures, I shall again and again have to point out to you that it is to the minutiæ, the details of antiseptic surgery that we must look for success; and I would impress upon you strongly that the carrying out of these details is in your hands, and ask you to neglect no precaution, however trivial, by which you may help to prevent sepsis.

Antiseptic Methods.—The question now arises, How are we to prevent our wounds becoming septic; or, if they are already so, what means can we adopt of again making them aseptic?

To keep our wounds sweet or aseptic, then, we must prevent, as far as possible, the entrance of organisms of any kind. This, of course, is only possible when we have to operate through unbroken skin, because otherwise in all probability the mischief will have been set up before the case comes into our hands, and then our endeavours will have to be directed towards abolishing the septic process. This is done by a judicious use of antiseptic agents, external and internal, including the improvement of the health of the tissues.

As an example of a case in which we may hope to maintain asepsis all through, let us take some surgical operation in which there is unbroken skin to begin with—for example, excision of the mamma for cancer. In such an operation there are the following sources of septic contamination, each of which must be very carefully guarded against: 1, The atmosphere; 2, the patient's own skin; 3, the surgeon's hands; 4, the hands of the nurse and other assistants; 5, the instruments, sponges, dressings, etc. These several dangers, and the means by which they are to be obviated, will be treated of in their appropriate places; meanwhile, I must ask you to bear with me while I briefly describe the more important antiseptics in general use, because unless you are acquainted with their names, actions, advantages, and dangers, you cannot hope to be successful in applying them in your practice.

Antiseptic Agents.—These are very numerous. The principal ones, however, are lotions, powders, oils, and wools.

Lotions.—There are many antiseptic lotions now in use, and I do not intend attempting to enumerate, much less to describe, all of them. There are three, however, which are so universally used, and so efficient, that I shall say a few words about each of them; they are—1, carbolic acid lotion; 2, corrosive sublimate lotion; 3, boracic acid lotion.

(1) Carbolic Acid Lotion.—This agent is of much historical interest, being that which Sir Joseph Lister first used when he introduced the antiseptic system about the year 1865. It is a substance derived from coal-tar by a complicated process of distillation, and in its pure, strong form is in long white crystals. As a lotion, however, it is used largely diluted with water. One part of the acid is dissolved in nineteen parts of water, and forms a lotion, spoken of as "one in twenty carbolic." This is the strongest carbolic lotion in common use, and for most purposes it is too strong. As a rule, a lotion half that strength, spoken of as "one in forty," is what is used in ordinary surgical operations and dressings. It is common in large hospitals to have on the table two bottles, labelled respectively "Carbolic acid, 1 in 20," "Poison," and "Carbolic acid, 1 in 40," "Poison."

Sometimes, however, only 1 in 20 is kept, and the weaker lotion is made by diluting this with an equal quantity of water. The lotion is quite clear if pure, and should it be the least turbid impurities, which will irritate the skin, may be suspected.

Caution!—You must bear in mind that carbolic acid is very poisonous, and the bottle should always bear a prominent "Poison" label. Not only is it deleterious when taken by the mouth, but when large quantities are used at an operation, or when a carbolic dressing is applied over a large area, such as an ulcer or burn, it may be absorbed, and give rise to unpleasant symptoms, such as giddiness, nausea, and vomiting. Some people are peculiarly susceptible to the action of carbolic acid, so when using it you should always keep a sharp look-out on your patient, and if he show any of these symptoms this lotion should be discontinued, and some other used. Evidence that the lotion is being absorbed is often to be found in the urine of the patient, which is passed of an olive-green colour, and on standing becomes almost black. This in itself is not a dangerous condition, but should be accepted as a hint that the patient is susceptible to the drug, and as an indication for making a change.

Advantages.—1, Its cheapness; 2, its volatility; 3, it does not injure instruments or sponges.

Disadvantages.—1, Being poisonous by absorption; 2, being irritant to skin of some patients.

Uses.—1 in 15: For spray. 1 in 20: 1, To purify skin of patient; 2, to purify hands; 3, to purify sponges and instruments; 4, to prepare gauze; 5, to prepare dipped towels. 1 in 40: 1, For operation lotion; 2, for dressing lotion.

Preparations.—1, Liquefied carbolic acid; 2, glyceride of carbolic acid; 3, carbolic acid and soap suppository; 4, carbolic ointment; 5, carbolic oil (non-officinal); 6, carbolic wool (non-officinal); 7, carbolic gauze (non-officinal).

*(To be continued.)*

### Presentations.

On Friday, the 24th ult., Dr. Newman (surgical lecturer), on behalf of the nurses of the Western Infirmary, Glasgow, presented Miss Clyde (the matron) with a handsome lamp and shade as a token of the respect and esteem in which she is held by them. Miss Clyde, in a suitable manner, acknowledged and thanked the nurses for their beautiful gift.

Miss Mary E. Thomas was, on November 6th, presented with the Radford Medal by Dr. Walter, visiting physician and lecturer to St. Mary's Hospital, Manchester. The late Dr. Radford left a sum of money to provide this medal, which is awarded annually to the best lady student for proficiency in the theory and practice of midwifery. This is the third year Miss Thomas has tried for the honour, running the winner very close each time, and now finally achieving success.

### Words of Consolation.

### FOR THE AGED.

We have reached and passed the fulness of the year, and the fruits of the earth have been garnered for future use. There seems a hush and pause in the seasons, as "lingering summer's parting step delays" to give place to chill October. The brightness and purity of the air enliven the heart, while the brilliant tints of the trees, gorgeous in orange, crimson, and russet brown, make splendid the surrounding landscape, and gladden the eye with beauty. The song of the redbreast "wooing the stillness of the autumn day," blends sweetly with the "calm decay," and speaks to the soul of "peace divine."

Especially to the aged does this time come with the greatest power, for our Heavenly Father, in His love and mercy, brightens the end of life with great present blessings, and precious promises for the future; just as autumn adds charms to cheer the departing year.

To the young, in their health and vigour, age seems as if it must be sad and dreary; but it is not necessarily so, for the old have joys which come only to them. The race of life has passed them by, they desire no longer the turmoil and pleasures of the world, and, having tasted how gracious the Lord is, are content to wait till they are ripe for the Home to which our dear Lord has gone before to prepare a place for them. "In My Father's house there are many mansions for those who love Him. He says, if it had not been so I would have told you." In the meantime there is much to comfort and console the aged for the loss of strength and energy. They live again in their children's children, which are "the crown of old men," and lavish on them the love and interest of their declining years. Peace and calm rest upon them. Happy for them if they can say with the Apostle Paul, as he neared the time of his departure, "I have fought a good fight, I have finished my course; henceforth is laid up for me a crown of righteousness, which fadeth not away, which the righteous Lord will give me at that day, and to all them also that love His appearing."

It is a beautiful and glorious sight when the hoary head is found in the way of righteousness! The aged pilgrim is bright with the reflection of the glory for which he is longing, and, though cheerful and ready to wait for his Master's call, he rejoices nightly as he lays his head on his pillow that he is "a day's march nearer home."

To some, perhaps, who have had cares and sorrows more deep and bitter than most men, the thought that—

> Be the day dreary, or be the day long,
> At length it weareth to evensong, comes with relief. They have borne the heat and labour of the day, and are scarred with the conflict against sin and sorrow; they long to be at rest. Well, Christ offers it to you, my brother, in the words, "Come unto Me all that labour and are heavy laden, and I will refresh you." How glorious! He is the Rest, and He is the Refreshing. He will take you to that place where there are no more tears, neither sorrow nor crying, for all those things will be done away. Do not rest, then, in simple resignation, but rejoice in the glorious hope of everlasting life.

Some may say, "I have only just found Christ. How will He take me in after having spent my life in wandering from His fold?" If you have really found Him, be not afraid. The labourer who had been hired at the eleventh hour into God's vineyard was paid the same as him who had gone in at the third hour. There is joy among the angels over every sinner that repenteth; so be of good cheer, thy sins are forgiven thee, saith He who lieth not.

### Death in Our Ranks.

On October 29th, at S. E. Hospital, Nurse Harris, of enteric fever, caught while acting as assistant nurse in the Male Enteric Ward. She had been at the hospital nearly two years, and intended shortly to leave for Plymouth. This makes the sixth death in the staff of this hospital since last Christmas Day.

### Nurses on Their Travels.

### NOTES FROM AUSTRALIA.

MELBOURNE, SEPT. 16TH.

Here is a hint for your Lords' Committee: On September 6th our Charities Commissioners paid a "surprise visit" to the Benevolent Asylum. That the institution is too small for the number of inmates it is called upon to accommodate was only too evident to the Commissioners. Every inch of room was occupied, and conditions of health—putting aside all the questions of comfort and convenience—demand some extension. Otherwise the Commission has been chiefly engaged with the subject of the necessity for a foundling hospital. Mr. Leslie, of the Eye and Ear Hospital, said it had been urged that the establishment of a foundling hospital would be conducive to immorality. Such a theory was a libel upon womanhood. He considered that the stimulating dietary of our young people—so ill-adapted to our climate—was a much more potent factor in that direction than a foundling hospital could possibly be. He imagined that comparatively few young women allowed themselves to lapse from virtue deliberately. So far the witnesses have been in favour of a foundling hospital. There has been a clean sweep at Melbourne Hospital of all the old rules and regulations, and a House Committee has been appointed to see that the new rules are kept. There are explicit definitions of the duties of sisters, nurses, and probationers.

Melbourne is now the proud possessor of a Dental Hospital, which was opened this week by Sir James MacBain.

Dr. Springthorpe, president of the Dental Association of Victoria, proposed "The New Dental Staff." Dentistry, as a profession, might be said to have been inaugurated officially by the Dentists Act, 1887. Since then the Dental Board had registered some 500 dentists, and it was through the Dental Association of Victoria that that hospital was founded. Necessitous persons suffering pain would be treated at once, without any subscriber's note whatever, and every provision was made for the prompt and skilful treatment of the patients.

A correspondent of the *Argus* has written a letter in favour of the paying wards of the Alfred Hospital. He says:—"The nature of my complaint—paralysis of both arms and legs—subsequent to an attack of influenza, necessitated an unusual amount of time and attention on the part of the nurses, more especially during the first month of my illness. Under the treatment of massage, use of the galvanic battery, injection of strychnine, &c., as recommended by the honorary surgeon and the resident doctor, I am happy to attribute (with God's blessing) my unexpected recovery, and it affords me much pleasure to testify to the careful, cheerful, and kind attention I received from all the nurses who waited upon me. Of the skilful treatment of the resident doctor and the courtesy and kindness on the part of the matron, Mrs. Strong, I cannot speak too highly." By-the-bye, speaking of massage, there is a perfect rage here on the subject; every other door bears the brass plate of masseur or masseuse, and everyone is being "massed." The other day a politician went to a masseur for a course of treatment, saying he had a big speech to deliver in a few days and he wanted "to get suppled up for it."

In "Notes from Australia" last month, mention was made of the outbreak of diphtheria in South Melbourne. Four children and the mother died, and a nurse was very ill. As the season advances more cases are heard of, both in the city and in the country. This month there has been a bad outbreak of typhoid at one of our prettiest and wealthiest suburbs, viz., Toorak. The houses are large, many of them on high ground with loveliest gardens surrounding them. The milk supply has been faulty. As the hot days come we shall be having more typhoid. Two ladies, well known in the nursing world in Melbourne, are seeking a suitable house in which they will take typoid patients and treat them with the greatest skill that is to be had here. Such a private hospital is greatly needed. We will give further particulars as soon as their arrangements are complete Another need is being met by the recent establishment of a dental hospital in the city.

Miss Sadler, known in hospital as Nurse Margaret, has. recently been appointed "Sister-in-Charge" of the men's ward in Launceston Hospital, Tasmania. She has been trained in the Women's Hospital, Melbourne. She holds certificates from both. In the Alfred Hospital she was assistant nurse, and lately relieving head nurse.

Of course the strike affects us: the hospitals have had to get in candles in case of failure of gas, and the coal contract of some hospitals has failed. If any of you are thinking of sending over help for the strikers—don't; keep it for your English charities. There is a good picture in the *Australasian* of a fat Colonial begging from some half-starved Irish peasants.

### Lectures.

THERE are some excellent lectures being given at the Institute of Medical Electricity which must interest nurses. In his third lecture on Masso-Therapeutics, Dr. Harries explains the courses of the arteries and veins, commencing with the pulmonary system, and directing attention to the fact that, contrary to the rule, the pulmonary artery contains impure and the veins pure blood. He then traced the circulatory vessels throughout the body, illustrating his remarks by large diagrams. The lecturer dwelt on the important part played by massage in helping the veins to perform their office of collecting and returning the impure blood to the heart, and pointed out that the venous system lay near the surface of the body, and was, therefore, conveniently placed for the hand of the manipulator. The explanations were clearly given, and to the point. It would be well if those of our practising masseuses who have hitherto been superior to anatomy could attend these lectures.

At the same institute a course of lectures on electro-therapeutics is being given. In his discourse on batteries last week Mr. Lawrence illustrated, by means of practical experiments, the difference in the currents of electricity generated by various machines. By connecting in parallel a series of four or more cells, a constant or continuous current was obtained. The lecturer said he preferred the word "continuous," for, as those who were accustomed to these batteries well knew, the strength of the current was affected by the condition of the weather and the freshness of the charging. The size of the cells made little difference to the electro-motive force or power of overcoming resistance. In the coil battery an interrupted or primary current was obtained by means of a bar of magnetized iron, which attracted and caused to vibrate a small piece of metal attached to a spring. The secondary or induction coil had no connection with the primary, though its current was set up by the neighbourhood of the latter. The secondary coil altered the quality and increased the strength of its borrowed electricity; the current was zigzag or alternating, and the electro-motive force was enormously greater. A patient who would find little inconvenience from the constant current could not possibly bear the same amount of current force applied through a coil. The secondary coil might be compared to a gossip who, having heard a slander, spreads the story with alterations and additions, though in the case of medical electricity these changes were not for the worse. In magneto machines a series of currents, similar to the secondary, were induced by moving or whirling a wire between two fixed magnets; these batteries were not good for medical practice, as the electricity was set in action by turning a handle, and it was difficult to keep up a perfectly even movement; inequality in the turns was painful to the patient. Mr. Lawrence offered to supply samples of magneto-electricity to his audience, but the offer was not eagerly accepted.

### Everybody's Opinion.

[*Correspondence on all subjects is invited, but we cannot in any way be responsible for the opinions expressed by our correspondents. No communications can be entertained if the name and address of the correspondent is not given, or unless one side of the paper only be written on.*]

### MALE NURSES.

THE SECRETARY OF THE HAMILTON ASSOCIATION writes: The statement of "Peckham" in your issue for November 1st is an absolute falsehood. No nurse has ever been dismissed for the reason stated. Two nurses voluntarily withdrew because the caution money was increased, and another subsequently resigned in consequence of the rules being altered; two out of the three afterwards applied to be re-admitted, and were allowed to re-join.

### THE TYNEMOUTH INFIRMARY.

IN the "Hospital Nursing Mirror" of November 1st., 1890, I notice a paragraph commenting on the management of the Tynemouth Infirmary. As it is evident to me that you must have been grossly and wilfully misinformed on certain details, I must ask you in common fairness to insert this letter. You state in your paper that "The Committee is composed chiefly of working men, and, strange to say, they seem to consider the comforts of the patients less than do those who have never had experience of illness in hospital wards. The utmost economy is observed in all departments. . . . The probationers have resigned, and all attempt at nurse-training given up." This I emphatically contradict. The committee of the Tynemouth Infirmary is composed of gentlemen from all classes, who have done everything in their power for the comfort and welfare of the patients. The "utmost" economy is most certainly not observed, as the diet of the patients is a generous one, and every luxury or nourishment prescribed by the medical staff is supplied without a murmur. The probationers were dispensed with by the Committee, as it was thought that they were of little practical use, but on the other hand caused a most serious drain on the resources of the institution. The Infirmary is now admirably managed and is doing good work. The nursing staff is excellent, and affords complete satisfaction to the Medical Board. I regret that you should have allowed your paper to have been the means of disseminating so unjust a criticism, and can only attribute the occurrence to misleading information.

I shall have great pleasure in forwarding to you a copy of the next Annual Report of the Tynemouth Infirmary, a perusal of which will substantiate my statements as to the good work done by, and the satisfactory management of the Institution. Thanking you for the insertion of this letter.—

ERNEST BRUMWELL, M.D.
Hon. Surgeon to the Tynemouth Infirmary.
5, Dockwray Square, North Shields.

### THE "SPECIAL PRO'S" ARTICLES: A PROTEST.

WILL you pardon me and others that we are glad to hope that there are no more letters from a "Special Pro." Nothing is to be gained in reading such letters, and they are not worthy of your excellent paper, THE HOSPITAL. It is painful to think that an accepted probationer could in such a flippant manner describe mental and physical suffering, life and death. There are many maladies too heartrending to be handed to the public by an inexperienced person. In such a glib manner is death spoken of that one wonders whether "Special Pro.'s" nursing tended more to that than life. I have read these letters again, and decide that I would not wish to be nursed by a woman who can so express herself, because there is a want of heart culture which no technical knowledge can give. The "bobbies," and not the "cases," seem to have most of "Special Pro's" sympathy.

ESTHER BAYLEY.
Paris.

### NORFOLK AND NORWICH HOSPITAL.

Mr. Howard I. Collins, the Secretary, writes :—A paragraph in the "Nursing Mirror" of this date, headed "Nurses at Norwich," seems likely to lead to the belief that the Special Committee appointed by the General Board of Governors is considering the "scheme for providing trained nurses for sick persons outside the hospital" as a new venture. The fact is, a staff of fully-trained nurses have for several years been sent out by this Hospital for private nursing, and it is a still further development of this scheme which is now being discussed. The earnings for the services of nurses last year amounted to £324 8s., and the receipts for the nine months of this year have already exceeded that amount. We are not, therefore, putting up a new staff in opposition to the Bethel Street Home, but extending our present staff. Overtures have been made with a view to amalgamating the two institutions, but it does not appear at present, to be likely to come about.

### LADIES ON COMMITTEES.

A. E. S. writes : I am an ardent admirer of Miss Twining, and always think she writes justly and sensibly on whatever subject she takes in hand. But I cannot agree with her as to women being on hospital committees. I think a sensible matron, well up in her work of housekeeping, cooking, and general management, would not submit to advice and interference from women, most of whom would know very little about it, and nurses and patients would have more complaints than they have already. The question of "ladies to help the matron"—save the mark !—was once mooted where I was working, and the senior of the honorary staff said, "When they come in, I shall go out." Thank goodness, they did not come in, so he is "on the staff" still. Hospital housekeeping is perfectly different in all ways to a private house, and wants as much training for it as does nursing. A matron must have a hard time always to keep things smooth, and it would not add to her comfort with her servants to have other mistresses poking about the larder and kitchens. Practical, working ladies are few and far between, and in a large hospital there is always "a housekeeper," and in a small one the matron (if the right woman) is sufficient.

### Competitions.

### SOMETHING NEW.

We have received over 60 answers to the Examination Question set last month, and shall next week announce the name of the nurse who excels some 59 of her fellows in knowledge and clearness of expression with regard to the precaution necessary in nursing typhoid cases. But, meanwhile, since our readers so highly appreciate competitions, we feel bound to offer something new in this direction. Therefore, the editor announces with pleasure that prizes of £2 and £1 (either in money or books, as the winners may prefer) will be given for the best bed-jacket or nightingale for an adult invalid received by December 17th. The work, comfort, and convenience of the bed-jacket will be considered in awarding the prizes, *not* the quality of the stuff, though we hope those nurses who can afford it will use woollen material, as the jackets will be sent to the hospitals as Christmas gifts from nurses. This competition should prove what ingenuity nurses possess in arranging for the comfort of their patients, and it ought to prove very acceptable to private nurses, who can employ long night watches in designing and making something which shall far surpass the present favourite nightingale. The articles sent in for this competition must be addressed "Hospital Competition, The Hospitals Association, Norfolk House, Norfolk Street, Strand, London," and must reach the Hospitals Association by December 17th. Carriage on all parcels must be prepaid. Every parcel must contain the full name and address of the sender, and a statement that it is her own work. And while on this subject we should like to state that there are about half-a-dozen duplicate dolls which we propose, unless the dressers object, to send to some children's hospital at Christmas.

### Vacancies.

**The following vacancies are announced :**

**Matron.**

Cancer Hospital, Brompton (Assist.)

**Superintendent.**

Hospital for Children, Great Ormond Street; 100 gs.

**Nurses.**

Ashton-under-Lyne Infirmary; £24 10s.; (night) £20.
Burton-on-Trent Institution; £25.
Belford Hospital, Fort William, N.B ; £30.
Bromley Nursing Institution.
Bedfordshire Institute, Bedford.
Bristol Hospital for Children and Women.
City and County Institution, Worcester.
Chester Diocesan Deaconess Institution ; £24.
Chester Union; £25.
City of London Chest Hospital, E.; £20.
Dorset County Hospital, Dorchester (night); £22.
Easter Ross Combination, Tain; £30.
Fulham Union (night); £20.
General Institute, Covent Garden.
Grosvenor Hospital, S.W.
Glamorgan and Monmouthshire Infirmary; £24.
Guest Hospital, Dudley (night); £22.
Hanover Institute, W.; £25.
Hampshire Institute, Southampton.
Huddersfield Home.
Hospital for Diseases of the Heart, Soho.
Jersey Institute; £25.
Leicester Institution: £25.
Middlesboro' Fever Hospital.
Manchester and Salford Sick Poor Institution; £25.
North London Consumption Hospital: £23.
North-Western Fever Hospital, N.W.; £30; Assistant, £22.
Nottingham and Notts Institution; £25.
Nurses' Home, Hull.
Preston Home; £20.
Royal Albert Edward Infirmary, Wigan; £25.
Rotherham Hospital; £20.
Salop Infirmary, Shrewsbury; £25.
Stephen Monkton Home.
St. Asaph Union; £25.
St. Leonard, Shoreditch; £20; assist., £17.
Sheffield Union; £20.
Samaritan Free Hospital, N.W.
St. Saviour's Infirmary, Dulwich, S.E.; £20.
South-Eastern Fever Hospital, S.E; £30.
Victoria Home, Bournemouth; £25.
Victoria Institute, Taunton; £18.
Workhouse Infirmary Association.
Wilson Institution, W.; £30.
Worthing Institute; £25.
Weston-super-Mare Hospital.
Warehousemen, Clerks, and Drapers' School, Purley; £25.
Wolverhampton Fever Hospital (Assist.)

**District Nurses.**

Stoke-on-Trent Home.
Durham Hospital Samaritan Society
Poplar and Stepney Sick Asylum, Bromley (night); £21 10s.

**Probationers.**

Beccles' Hospital.
Croydon General Hospital.
Coventry Fever Hospital.
East Grinstead Cottage Hospital.
Liverpool Hospital for Women.
Lloyd Cottage Hospital, Bridlington.
St. Leonard, Shoreditch; £10.
Sheffield Union; £10.
Workhouse Infirmary Association.
Welsh Central Home.

### Notes and Queries.

**Queries.**

(5) *Candidates at Isle of Wight.*—Will any lady who lately attended as a candidate for the post of lady superintendent kindly inform me whether she was asked if she was a member of the B. N. A.; and whether, if when she answered in the affirmative, things began to look black?—*Unsuccessful.*

(6) *Nurses' Cloaks.*—Where is the best place to get a nurse's cloak, and what do they cost—cloak to be half fitting and for winter wear?—*Lincoln.*

(7) *Home at Brighton.*—Particulars wanted at once of the home now open at Brighton for ladies recovering from illness.—*C. D.*

**Answers.**

*M. Donelly.*—Your letter has been forwarded.

*Registration.*—We beg to inform Mrs. N. that we are not in the least opposed to the principle of registration; witness the fact that we have persistently upheld the Midwives' Registration Bill. But this register would only include midwives who held diplomas, whereas the nurse registration scheme would include any so-called nurse who had been engaged in tending the sick for three years, and who held no certificate, no connection with any recognised training school at all. Cannot Mrs N. see the difference?

*The Case of the Older Nurses.*—We cannot forward all the letters for "Nightingale Sister" which have been sent us, but we have asked sister to write us an account of the duties of the trained nurse in the nursery, and we hope to take steps shortly to bring nurses into contact with the ladies who might thus appreciate their services.

*J. R. R.*—Sorry our poets are becoming so numerous; we are obliged to refuse all verse for the present.

*London Hospital Nurses.*—We are much obliged for the many letters in favour of this institution sent us, but we think enough has been printed to prove that the grumblers are only a very small section, and that the public knows how much credence to give to their cries.

*Postcards from Paris.*—The writer of these anonymous postcards is warned that her name is known, also the absence of truth in her statements.

*F. M. B.*—We shall not publish your letter, as we have made inquiries and found it incorrect.

(3) *Nettle Rash* is caused through having eaten some indigestible food, especially shell fish; and in infants by the impoverished and disordered condition of the mother's milk. The rash generally disappears in the daytime and returns in the evening, in this manner often continuing for several days, or even weeks. It finally disappears by desquamation.—*Betha.*

*A. E. G.* must send her full name.

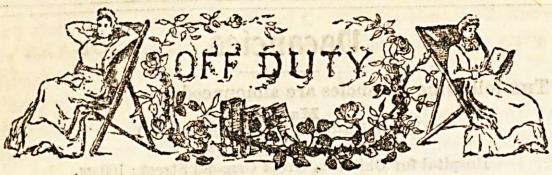

### An Eventful Night.

We of the nursing sisterhood have, as a sequence of the intimate relationship with people's inner natures into which sickness draws us, a store of strange episodes laid by in our memories. Some of these incidents are full of pathos; others are as full of horror. Were our dealings with fiction instead of reality, the world of readers would undoubtedly be the richer. Of the many occurrences which go to form my own experience there is one never far off from my thoughts.

On a certain night I was on duty in a male ward on the top floor of a London hospital. My ward was at some distance from the others, and the building was a large one, the blocks being connected by bridges. When I went on duty that night, I was told by the day nurse that a new patient had been admitted to my ward. There was nothing, of course, unusual in that fact, for we had fresh patients brought in nearly every day. This particular one was supposed to be suffering from bronchitis; when I went to his bedside I found him apparently asleep, but muttering a good deal to himself. Not considering that a symptom of any great importance, I went my rounds, and then repaired to the little kitchen of the ward. While there a sudden noise attracted my attention, and returning hastily to the ward I found my new patient sitting up in bed fighting some imaginary foe. His eye turned upon me instantly, and gleamed with such frenzy that a quick conviction of the man's insanity rushed over me.

The ward was full of men, but most of them were bad cases, and not any one of them capable of rendering me assistance should I be in need of such. Cautiously closing the room door, I looked around to see if there were anything within reach likely to prove dangerous. I espied the poker. But so did my patient! Comprehending, with a curious alertness of mind, my intention of making for the weapon, he, in one bound, reached it before I could.

For that time he was the victor; I the vanquished.

Then, to the sickening horror of myself and the helpless spectators, began a chase round the ward, the new-comer, armed with the formidable poker, pursuing me furiously. In the middle of the ward were two stoves, by dodging round which I managed to put as great a distance as possible between my crazed pursuer and my hapless self.

"Run out of the ward, nurse! Get outside, and save yourself!" feebly implored the rest of the terror-struck patients with one voice.

But no! "England expects every" nurse, as well as every "man, to do their duty," and I was determined, with God's help, to do mine that night; so on sped the terrible race. After some time, however, the strength of my hunter began to flag; he drew up, suddenly; then, languidly and quietly, went, of his own accord, straight back to his bed, and lay down.

Controlling my nervous feelings with a violent effort, I waited until the ward became hushed in silence, and the men all slept. Then I asked myself, Would it be safe to cross the bridge and touch the alarm-bell which was there? Dare I leave my poor, helpless patients with a madman? Fearing to do so, I, for the moment, went back to the kitchen, in order to make up the fire, which had nearly died out. Occupied thus I seemed to be aware that a figure flitted past the open door. Telling myself it was pure imagination, I, however, went quickly outside, and found it to be a ghastly reality. My dreadful patient was slipping quietly through the door leading to the bridge! The shock almost caused my heart to stop beating; the man was tall and powerful, and, though I was strong, what would my strength be but puny if meted against such as his? Nevertheless, I flew after him to the spot where he stood gazing down into the awful depth below. Just as he pulled himself together to take the mad leap into space, I seized him, pulling him back by main force. As I did so, I screamed loudly for the help so sorely needed, but the night was stormy, and the howls and moans of the wind drowned my voice. I felt, with a swift conviction, that if assistance did not instantly come from somewhere, either my patient or myself, perhaps both, would be hurled over the bridge, to a certain death below. But I dared not leave him. If I could only persuade him, by some strategy, to go towards the door at the other end of the bridge, I should then be able to snatch at the bell, the ringing of which would bring instant help. It was my only chance—and his—for life!

"Look!" I whispered hoarsely, as I pointed to the door, "That's the way down! That's the way!"

The maniac hesitated for what seemed to me an eternal moment. I trembled, with an overpowering terror, as I waited for his decision. Would he believe me, and go down by the door, or would he go by his own road—over the fatal bridge, dragging me with him?

With a sudden darting motion, he rushed forward to the door, I following at his heels. We were saved! Scarcely had I touched the alarm-bell when the porter appeared, and danger was past.

This narrow escape of a dire tragedy had been enacted without anyone in the hospital hearing a sound. There was no doubt as to the insanity of the unfortunate creature, who was next day removed to his proper place—an asylum. As for me, praises were showered upon me for the courage I had displayed. But the price of that courage was a heavy one; weeks of illness laid me low, and when I crept back to health and life, it was with perfectly snow-white hair.

### Amusements and Relaxation.

### N.B.-THIRD QUARTERLY WORD COMPETITION

**Commenced Oct. 4, 1890; ends Dec. 27, 1890.**

Three prizes of 15s., 10s., 5s., will be given for the largest number of words derived from the words set for dissection.

Proper names, abbreviations, foreign words, words of less than four letters, and repetitions are barred; plurals, and past and present participles of verbs, are allowed. Nuttall's *Standard* dictionary only to be used.

N.B.—Word dissections must be sent in WEEKLY not later than the first post on Thursday to the *Prize Editor*, 140, Strand, W.C., arranged alphabetically, with correct total affixed.

The word for dissection for this, the SIXTH week of the quarter, being "AMBULANCE."

| Names. | Oct. 30th. | Totals. | Names. | Oct. 30th. | Totals. |
|---|---|---|---|---|---|
| Jenny Wren | 62 | 187 | Liz | 43 | 126 |
| Tinie | — | 55 | Checkmate | — | 76 |
| Agamemnon | 66 | 188 | Silver King | 65 | 163 |
| Patience | 67 | 187 | S. Anthony | — | 76 |
| Ecila | 68 | 187 | Quackah | — | 75 |
| Lightowlers | 65 | 180 | Reynard | 61 | 149 |
| Rouge | — | 89 | Sally | — | 27 |
| Wyamaris | 67 | 172 | Success | — | 61 |
| Qu'appelle | 61 | 166 | Caledonia | — | 52 |
| Nosam | 60 | 167 | Nurse Emma | 28 | 94 |
| Nurse Hilda | — | 44 | Hazel | — | 20 |
| Lady Betty | 67 | 171 | Pallas | — | 48 |
| Grenelle | — | 43 | Puss | — | 15 |
| Daisy | 59 | 155 | Shakespear | 48 | 115 |
| H. A. S. | 49 | 142 | Melita | 65 | 170 |
| A. B. C. | — | 66 | | | |

**Notice to Correspondents.**

N.B.—Each paper must be signed by the author with his or her real name and address. A *nom de plume* may be added if the writer does not desire to be referred to by us by his real name. In the case of all prize-winners, however, the real name and address will be published.

Competitors can enter for *all* quarterly competitions, but no competitor may take more than *one first prize* during the year.

